# Characteristics of High Suicide Risk Messages From Users of a Social Network—Sina Weibo “Tree Hole”

**DOI:** 10.3389/fpsyt.2022.789504

**Published:** 2022-02-18

**Authors:** Bing Xiang Yang, Pan Chen, Xin Yi Li, Fang Yang, Zhisheng Huang, Guanghui Fu, Dan Luo, Xiao Qin Wang, Wentian Li, Li Wen, Junyong Zhu, Qian Liu

**Affiliations:** ^1^School of Nursing, Wuhan University, Wuhan, China; ^2^Department of Psychiatry, Renmin Hospital of Wuhan University, Wuhan, China; ^3^Population and Health Research Center, Wuhan University, Wuhan, China; ^4^Division of Mathematics and Computer Science, Faculty of Sciences, Vrije University Amsterdam, Amsterdam, Netherlands; ^5^Department of Information Science, Beijing University of Technology, Beijing, China; ^6^Affiliated Wuhan Mental Health Center, Tongji Medical College of Huazhong University of Science & Technology, Wuhan, China; ^7^Department of Nursing, Renmin Hospital of Wuhan University, Wuhan, China; ^8^School of Public Health, Wuhan University, Wuhan, China

**Keywords:** suicide, tree hole of Weibo, artificial intelligence, social media, content analysis

## Abstract

**Background:**

People with suicidal ideation post suicide-related information on social media, and some may choose collective suicide. Sina Weibo is one of the most popular social media platforms in China, and “Zoufan” is one of the largest depression “Tree Holes.” To collect suicide warning information and prevent suicide behaviors, researchers conducted real-time network monitoring of messages in the “Zoufan” tree hole via artificial intelligence robots.

**Objective:**

To explore characteristics of time, content and suicidal behaviors by analyzing high suicide risk comments in the “Zoufan” tree hole.

**Methods:**

Knowledge graph technology was used to screen high suicide risk comments in the “Zoufan” tree hole. Users' level of activity was analyzed by calculating the number of messages per hour. Words in messages were segmented by a Jieba tool. Keywords and a keywords co-occurrence matrix were extracted using a TF-IDF algorithm. Gephi software was used to conduct keywords co-occurrence network analysis.

**Results:**

Among 5,766 high suicide risk comments, 73.27% were level 7 (suicide method was determined but not the suicide date). Females and users from economically developed cities are more likely to express suicide ideation on social media. High suicide risk users were more active during nighttime, and they expressed strong negative emotions and willingness to end their life. Jumping off buildings, wrist slashing, burning charcoal, hanging and sleeping pills were the most frequently mentioned suicide methods. About 17.55% of comments included suicide invitations. Negative cognition and emotions are the most common suicide reason.

**Conclusion:**

Users sending high risk suicide messages on social media expressed strong suicidal ideation. Females and users from economically developed cities were more likely to leave high suicide risk comments on social media. Nighttime was the most active period for users. Characteristics of high suicide risk messages help to improve the automatic suicide monitoring system. More advanced technologies are needed to perform critical analysis to obtain accurate characteristics of the users and messages on social media. It is necessary to improve the 24-h crisis warning and intervention system for social media and create a good online social environment.

## Introduction

Suicide is an important social issue and has become the second leading cause of death among 15–29 years old globally (1). In China, the suicide rate is 0.0097% (2). Early identification of people with suicide risk is crucial for suicide prevention. However, these people usually do not actively seek help, so traditional methods such as self-reported ratings and structured interviews are ineffective in identifying suicide risk in time. Nowadays, because of the large number of Internet users and the anonymity of the Internet, people tend to express their negative emotions, even suicidal thoughts and plans through social media. Posting suicide or self-harm information on social media is regarded as a signal of suicidal ideation, and it may increase the contagious effect of suicidality since suicidal behaviors may be learned from others (3–5). Therefore, the association between suicide and social media has become a public health concern (6).

The words people used on social media are important cues to their mental health status. Due to a large number of posts and comments on social media, it is hard to identify and analyze suicide-related texts manually. In recent years, many machine learning methods were used for text sentiment analysis. A review showed that natural language processing and information retrieval methods were frequently used to extract language characteristics and predict future incidents of suicide or suicide attempts (7). In China, studies analyzing suicide-related text on social media were mainly based on Sina Weibo, the most popular microblog with a 42.3% utilization rate in China (8). A study used deep learning methods to build a text classifier to identify users on Sina Weibo with depression and negative emotions, and it found users with depression were more active than general users, and they expressed hopelessness or sadness, discussed depression treatment, suicide or self-injury (9). Another study showed that the Simplified Chinese-Linguistic Inquiry and Word Count (SC-LIWC) dictionary and machine learning method were useful to automatically identify language markers of suicide risk or emotional distress of users on Sina Weibo (10), and a higher usage of pronouns, prepend words (mainly preposition), multifunction words and a lower frequency of verb usage in messages, and a greater total word count were associated with a higher suicide possibility (10). Sina Weibo users who ended his or her life interacted less with others, had a higher level of self-concern, and used more negative expressions, more religious and death-related words, and less work-related words (11).

Huang developed the “Tree Hole Intelligent Agent” by using Knowledge Graph technology to automatically identify users with different levels of suicide risk in the “Zoufan” tree hole (12). “Zoufan” is one of the biggest “Tree Holes” on Sina Weibo. On March 17, 2012, a Sina Weibo user named “Zoufan” posted her last tweet: “I suffer from depression, so I just choose to die, for no important reason. Don't worry about my death.” After her death, her last post attracted many people to express their negative emotions and became a “Tree Hole.” To date, there are more than 2 million messages from 350,000 users commented under her last post which share suicidal thoughts and plans. Some users even sent suicide invitations to implement collective suicide. The analysis of general comments in the “Zoufan” tree hole showed that 52% of comments tended to be negative; emotional expression, relationships and social support, sleep and death were high-frequency keywords mentioned in messages (13). Huang's “Tree Hole Intelligent Agent” classifies the suicide risk of “Tree Holes” users according to the certainty of suicide methods and the urgency of time mentioned in their comments. High suicide risk messages refer to those including suicide plans or indicating users may commit suicide soon. High suicide risk users are people who send high suicide risk messages. The “Tree Hole Action” started by Huang provides proactive suicide crisis intervention for high suicide risk users, and it has temporarily prevented 3,629 potential suicides from 2018 to 2020 (14).

High suicide risk users on social media should be the focus of suicide prevention because they are most likely to commit suicide and need timely crisis intervention. However, current studies are not enough to provide a clear portrait of high suicide risk users on social media. In addition, most studies analyzed posts on users' home pages rather than their comments under others' posts. The latter is more difficult to be found by familiar people, so it may provide a better understanding of the inner world of users with suicide risk. The comments section is also interactive because users not only express themselves but also discuss with others.

Therefore, this study analyzed the high suicide risk comments under the last post of the “Zoufan” tree hole to explore the characteristics of users and high suicide risk messages. The findings can provide insights into the portrait of high suicide risk users on social media, help to improve the performance of “Tree Hole Intelligent Agent,” and facilitate the development of early suicide monitoring and proactive crisis intervention such as “Tree Hole Action” intervention through artificial intelligence technology. The findings also provide evidence for developing targeted long-term support programs on social media for high suicide risk users.

## Methods

### Data Collection

“Tree Hole” AI robots were used to crawl users' messages in the “Zoufan” tree hole from November 6, 2018 to May 5, 2020. According to the certainty of suicide manner and the urgency of the time, a suicide risk rating was established by using Knowledge Graph technology (12). The Knowledge Graph is a graph-based knowledge representation and organization method, which can represent systematic, structured, and integrated domain-specific knowledge based on semantic technology (12, 15). The suicide risk classification standards are as follows (12): level 10 (suicide may be in progress), level 9 (suicide method has been determined and may occur soon), level 8 (suicide has been planned, and the suicide date is generally determined), level 7 (suicide method has been determined, and the suicide date is unknown), level 6 (suicide has been planned, and the suicide date is unknown), level 5 (expression of strong desire to commit suicide, and the suicide method is unknown), level 4 (suicidal desire has been expressed, and the specific method and plan are unknown), level 3 (intense survival pain, and no suicidal wishes expressed), level 2 (survival pain has been clearly expressed, and no suicidal wishes expressed), level 1 (survival pain is partially expressed, and no suicidal wishes expressed), and level 0 (no expression of survival pain noted). Messages were graded automatically by AI robots, and levels from 6 to 10 were marked as high suicide risk messages. The accuracy of the identification of suicide risk has reached 82% (12).

### Data Analysis

The age, gender and region of Sina Weibo users were described by frequency and percentage. The users' level of activity was analyzed by calculating the number of messages per hour.

Words in the messages were segmented by a Jieba tool. The keywords and a keywords co-occurrence matrix were extracted using a TF-IDF algorithm. Jieba tool is a method suitable for Chinese word segmentation, which can divide continuous word sequences into word sequences. TF-IDF (Term Frequency-Inverse Document Frequency) algorithm is a statistical method used to evaluate the importance of words in the text, and a weighted technology in information retrieval and data mining (16). Researchers divided high-frequency keywords into four classifications (suicide-related words, emotion expression words, role-relevant words and time and place relevant words) manually according to the contents. Keywords co-occurrence network analysis was constructed by Gephi 0.9.2 software. Each node represented a keyword; “degree” represented the frequency of the paired keywords that appeared together in each message; “edge” meant the connection between the paired keywords. The weight of the edge referred to the closeness of the paired keywords. The greater the weight of the edge, the closer the relationship between the two nodes.

To explore the suicide characteristics, researchers reviewed each message, manually annotated the suicide reasons, and identified if the users sent suicide invitations.

### Ethical Approval

This study received approval from the Ethics Committee of Wuhan University School of Medicine (code: 2020YF0075).

## Results

### General and Time Characteristics

In this study, there were totally 5,760 high suicide risk messages: 1,190 (20.66%) were level 6, 4,222 (73.30%) were level 7, 48 (0.83%) were level 8, and 300 (5.21%) were level 9.

**Figure 1:**
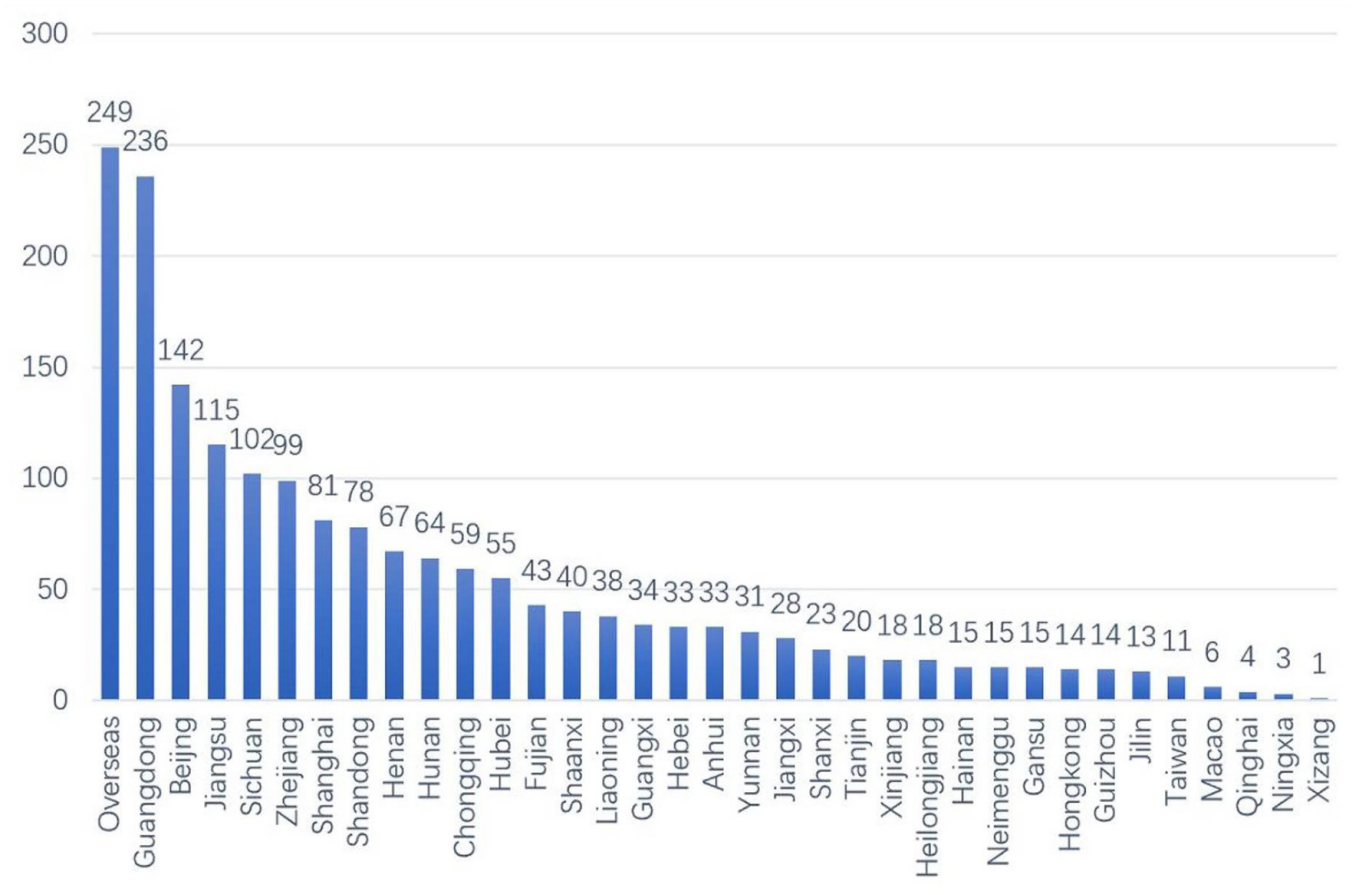
The geographic location of users.

According to users' registration information, 2,105 (64.79%) were female users, 762 (23.45%) were male users and 382 (11.76%) had no gender identified. Many users (1,818) reported a geographic location, including outside of China (249, 13.70%), Guangdong (236, 12.98%), Beijing (142, 7.81%), Jiangsu (115, 6.33%), and Sichuan (102, 5.61%), etc. (see Figure 1).

**Figure 2:**
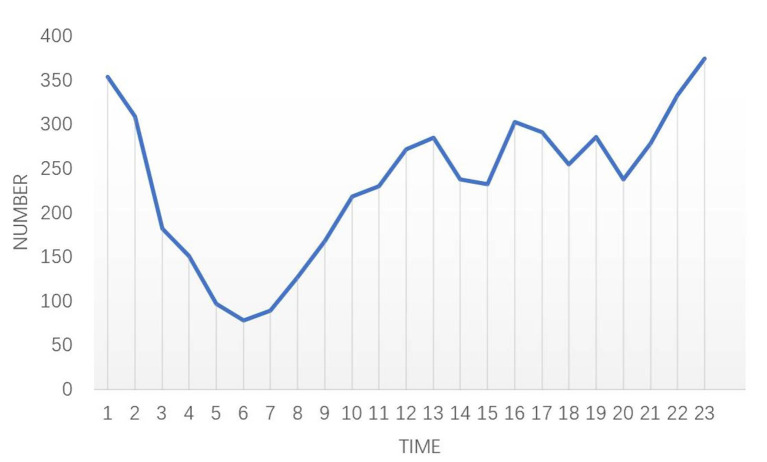
Characteristics of high suicide risk messages.

About 19.08% of messages were posted between 23:00 and 01:00, while the fewest messages (264, 4.58%) were posted between 05:00 and 07:00 (see Figure 2). The horizontal axis represents the time of the message posting and the vertical axis represents message volume per hour.

### Content Characteristics

The keywords extracted by the TF-IDF algorithm were sorted by weight. The weight is a statistical measure calculated by TF-IDF to evaluate the importance of a word in a text or corpus (17). The top 20 keywords with great weight are shown in Table 1. The top 50 keywords are shown in the Appendix.

**Table 1:** Characteristics of keywords (Top 20).

**Keyword**	**Weight (frequency)**	**Keyword**	**Weight (frequency)**
Jump off buildings	0.6644 (1662)	Leave	0.0646 (356)
Slash wrist	0.4353 (977)	World	0.0564 (380)
Burn charcoal	0.3406 (888)	Don't want	0.0559 (268)
Hang	0.1546 (385)	Have or not	0.0559 (224)
Really	0.1218 (592)	Alive	0.0463 (171)
Jump into a river	0.1178 (253)	Feel	0.0427 (200)
Jump down	0.1005 (286)	Uncomfortable	0.0378 (131)
Suicide	0.0920 (366)	Taking pills	0.0345 (109)
Pain	0.0840 (328)	Afraid of pain	0.0321 (69)
Sleeping pills	0.0729 (189)	Sad	0.0316 (121)

The high-frequency keywords were divided into four classifications, including suicide-related words, emotion expression words, role-relevant words and time and place-relevant words (see Table 2). Suicide-related words and emotion expression words were the most mentioned classifications. Jumping off buildings, wrist slashing, burning charcoal, hanging and sleeping pills were the most mentioned suicide methods.

**Table 2:** Classification of high-frequency keywords (unit: frequency).

**Suicide-related words**		**Emotion expression words**		**Role-relevant words**		**Time and place relevant words**	
Jump off buildings	1,662	Pain	328	Parents	125	School	54
Slash wrist	977	Uncomfortable	252	Mama	107	Hospital	49
Burn charcoal	888	Happy	121	Family	68	Roof	29
Hang	385	Dare not	120	Wardmate	50	Window	28
Suicide	366	Afraid	107	Kids	44	Balcony	21
Leave	356	Like	94	Girls	34	Early morning	21
Jump into a river/sea	293	Sorry	71	Classmate	27	Just now	77
Jump down	286	Afraid of pain	69	Doctor	24	A few days	62
Sleeping pills	189	Hahaha	69	Elderly sister	19	Recently	61
Death	86	Desperate	54	Daughter	18	Before	47
Relieve	73	Broken down	45	Boyfriend	17	Tonight	35
Self-harm	49	Regret	41			Last night	27
Posthumous papers	43	Dislike	39			Future	20
Hang to die	29	Very tired	31			Daytime	16
Pesticide	23	Sad	31			Afternoon	16

The keywords co-occurrence network analysis showed that “world-leave,” “jump off buildings-really,” “jump off buildings-slash wrist,” “jump off buildings-hang,” and “suicide-burn charcoal” were the top five co-occurrence relationships. The top five related keywords with “pain” were “jump off buildings,” “burn charcoal,” “slash wrist,” “hang,” and “really.” The top five related keywords with “happy” were “jump off buildings,” “really,” “jump down,” “alive,” and “burn charcoal” (see Table 3).

**Table 3:** Co-occurrence relationships of keywords (Top 10).

**Partial keywords**	**Co-occurrence keywords (frequency)**
Pain	Jump off buildings (228), burn charcoal (214), slash wrist (162), hang (128), really (124), very painful (108), not painful (80), alive (70), world (64), leave (54)
Uncomfortable	World (34), very painful (16), mama (16), can't stand (14), very tired (12), wake up (8), useless (8), afraid of pain (6), death method (6), parents (4)
Happy	Jump off buildings (78), really (40), jump down (22), alive (20), burn charcoal (18), sad (18), hope (16), world (16), like (14), feel (14)
Alive	Really (90), world (64), very tired (38), hang (36), want to die (36), burn charcoal (34), well (30), pass away (30), go to die (30), parents (26)
Suicide	Burn charcoal (254), jump off buildings (236), slash wrist (172), really (128), don't want to (80), depression (76), want to die (76), pain (64), world (56), sleep pills (52)

Both of the keywords and the keywords co-occurrence network analysis showed that high suicide risk users had a strong willingness to end his or her life, and they were concerned about suicide methods and expressed great negative emotions. It is consistent with many original posts. For example, “I searched many methods, jumping into a river, burning charcoal, taking medicine, jumping off the building. The highest success rate should be to jump off the building……”

### Suicidal Characteristics

Among all high suicide-risk messages, 1,011 (17.55%) included suicide invitations and 327 (5.68%) were automatically rated as high suicide risk by AI roots but didn't express definite suicide ideation, such as “No, friends. The world may not be worth living, but there are also some things in the world that you will cherish.”

There were different reasons for sending suicide invitations to others. Some users want to suicide with others because they have no courage or they want to have a companion: “It's lonely to die alone, is there anyone die with me?” “I thought that if two people died together, maybe I'll have more courage.”

Negative cognition and emotions such as sadness, desperation and meaninglessness, mental disorders and specific problems in life such as family issues were common suicide reasons in high suicide risk messages. Some users were not willing to seek help, for example, “I know I have been ill for a long time, but I am reluctant to spend money to see a doctor…” Some users expressed suicidal ideation but did not implement it because they were worried about their family: “I want to jump off the building, but I worried my parents will be sad, I have no courage.”

## Discussion

This study analyzed the characteristics of users with high suicide risk and their comments under the last post of “Zoufan” tree hole— one of the biggest “Tree Holes” in Sina Weibo. The study showed that females and users from economically developed cities are more likely to express suicide ideation on social media. High suicide risk users were more active during nighttime, and they expressed strong negative emotions and willingness to end their life. Jumping off buildings, wrist slashing, burning charcoal, hanging and sleeping pills were the most frequently mentioned suicide methods. The most common suicide reasons were negative cognition and emotions. About 17.55% of comments included suicide invitations. Some users did not commit suicide because they did not want their families to suffer because of their death.

### What This Study Adds to Current Literature

First, previous studies mainly analyzed the content characteristics of posts on users' home pages, but this study included the comments with high suicide risk that were publicly available under the last post of a girl who killed herself named “Zoufan.” Because the “Zoufan” tree hole is one of the biggest “Tree Holes” in Sina Weibo, these comments can help to provide a better understanding regarding the inner world of users with high suicide risk. Second, our previous study about the general comments under the last post of “Zoufan” showed that 52% of comments expressed negative emotion. By comparing the characteristics of general comments with high suicide risk comments, relationship-related keywords (e.g., “I love you,” “boyfriend,” “break up”) and sleep-related keywords (e.g., “can't fall asleep,” “insomnia”) were frequently mentioned in general comments (13), but these were not high-frequency keywords in high suicide risk comments. In contrast, time and place relevant words (e.g., “school,” “hospital,” “roof”) were high-frequency keywords in high suicide risk comments but not in general comments (13). Suicide-related words such as jump off buildings, slash wrist, burn charcoal were high-frequency keywords in both but were mentioned more frequently in high suicide risk comments than general comments (13). These linguistic features help to form the portrait of high suicide risk users on social media, which is crucial for suicide monitoring and intervention.

### Implications for Automatically Suicide Monitoring and Identification

The findings of this study provide evidence to improve the performance of the “Tree Hole Intelligent Agent,” which is developed based on knowledge graph technology (12). Ontology can be regarded as a specific type of knowledge graph (12). Now the “Tree Hole Ontology” has four parts: suicide ontology, time ontology, space ontology and desire ontology. This study showed that besides suicide-related words, time and place related words, emotion expression words and role-related words were also high-frequency words mentioned by high suicide risk users. Therefore, emotion ontology and relationship ontology can be added to improve the performance of “Tree Hole Intelligent Agent.” The results of content analysis in this study also contribute to the complement of “suicide dictionary” which can be used in suicide tendency analysis. The co-occurrence words help to enrich and expand the logical rules of knowledge-based methods (18), as domain knowledge to build deep learning based sentiment analysis algorithms (19).

### Implications for Mental Health Promotion Projects and Social Media Suicide Intervention Systems

The suicide crisis interventions require close collaboration among the government, society, social media platforms, healthcare professionals and the family. Government and the society should publicize mental health education and life education, help people to enhance positive coping skills, destigmatize mental illness and emphasize the importance of seeking help when necessary. Since expressing emotions is a protective factor of suicide (20), it is important to create a friendly and supportive environment for people to express their emotions. People with suicidal thoughts are more willing to seek help from mental health hotlines and the internet due to the convenience and anonymity (21), so the government, social media platforms and healthcare institutions could work together to set up mental health hotlines and online forums to provide professional consultations and interventions.

When building mental health service systems, the government and healthcare institutions should pay more attention to high-risk regions, actively publicize ways to cope with work and life pressure and focus on improving social support networks. Because high risk suicide users are more active at night, when healthcare providers deliver mental health education through social media, sending posts at night may be more effective (22). Due to the reduced availability of healthcare workers at night, it is necessary to develop automatic identification technology to enhance monitoring during the nighttime. Artificial intelligence technology could monitor the messages continuously and identify high suicide risk messages automatically. Since time is vital for suicide intervention, it is necessary to develop crisis intervention guidelines for suicide risk users on social media. Social media platforms can also introduce relevant policies like forbidding users to post messages including suicide invitations.

Family support is a crucial protective factor of suicide, and good family relationships can be a protective factor in preventing suicide (23). Therefore, the family needs to focus on the emotional status of their family members, notice early signs of suicide and seek professional interventions when necessary (24). Healthcare professionals should also incorporate family support and education into crisis interventions.

### Implications for Future Research

Because Sina Weibo has a 140-character limit for each comment, future studies could analyze users' comments in “Tree Holes” and posts on their home page together to get more comprehensive user portraits. The cross-cultural research of characteristics of suicide messages may also be implemented. Apart from the “Zoufan” tree hole, there are many other “Tree Holes” on Sina Weibo, so the automatic suicide monitoring model and intervention system developed for the “Zoufan” tree hole can be extended to other “Tree Holes.”

### Limitations

Firstly, messages in “Tree Holes” are fragmented and can't completely reflect the user's overall state. Secondly, because of the anonymity of online social media, the authenticity of messages and users' information can't be completely assured. Thirdly, using a software program to do the text analysis based on an identified algorithm may not accurately depict the characteristics of users' messages. For instance, sometimes software wrongly identifies the message advising others not to jump off buildings as a high suicide risk message. Future technology development can make up for this limitation, and software is expected to automatically identify and classify the cause of suicide to help researchers work more efficiently. In addition, classifications of keywords were based on keywords rather than deep semantics analysis of the complete text. More mature text semantic analysis technology can solve this problem in the future.

## Conclusion

Users sending high risk suicide messages on social media expressed strong suicidal ideation. Females and users from economically developed cities were more likely to leave high suicide risk comments on social media. Nighttime was the most active period for users. Characteristics of high suicide risk messages help to improve the automatic suicide monitoring system. More advanced technologies are needed to perform critical analysis to obtain accurate characteristics of the users and messages on social media. Future research should more pay attention to the mental health of social media users, improve the 24-h crisis warning and intervention system for social media and create a good online social environment.

## Data Availability Statement

The raw data supporting the conclusions of this article will be made available by the authors, without undue reservation.

## Ethics Statement

This study received approval from the Ethics Committee of Wuhan University School of Medicine (code: 2020YF0075).

## Author Contributions

BXY, PC, QL, JZ, and LW designed the study and wrote the research protocol. PC, XYL, BXY, QL, FY, JZ, LW, ZH, GF, DL, XQW, and WL did the literature review, managed the field survey, quality control, and statistical analysis and prepared the manuscript draft. QL, XYL, BXY, and FY contributed to the revisions in depth for the manuscript. BXY, QL, JZ, and LW supervised the survey and checked the data. All authors contributed to and approved the final manuscript.

## Funding

This study was supported by the grant from the Project of Humanities and Social Sciences of the Ministry of Education in China (The Proactive Levelled Intervention for Social Network Users' Emotional Crisis-an Automatic Crisis Balance Analysis Model, 20YJCZH204).

## Conflict of Interest

The authors declare that the research was conducted in the absence of any commercial or financial relationships that could be construed as a potential conflict of interest. The Handling Editor WV declared a shared affiliation, though no other collaboration, with one of the authors ZH at the time of the review.

## Publisher's Note

All claims expressed in this article are solely those of the authors and do not necessarily represent those of their affiliated organizations, or those of the publisher, the editors and the reviewers. Any product that may be evaluated in this article, or claim that may be made by its manufacturer, is not guaranteed or endorsed by the publisher.
