# Supplementary material for: Characteristics of High Suicide Risk Messages From Users of a Social Network—Sina Weibo “Tree Hole”
Source: Front Psychiatry. 2022 Feb 18;13:789504. doi: 10.3389/fpsyt.2022.789504 (PMC8900140; doi:10.3389/fpsyt.2022.789504)
Supplement: Supplementary file 1 [file Table_1.DOCX]

Appendix: Result of keywords (Top 50)

| Keyword | Weight  (frequency) | Keyword | Weight  (frequency) | Keyword | Weight  (frequency) | Keyword | Weight  (frequency) | Keyword | Weight  (frequency) |
| --- | --- | --- | --- | --- | --- | --- | --- | --- | --- |
| jump off buildings | 0.6644  (1662） | leave | 0.0646(356) | “Zoufan” | 0.0312(64) | courage | 0.0229(78) | parents | 0.0197(80) |
| slash wrist | 0.4353  (977） | world | 0.0564(380) | death method | 0.0298(76) | friend | 0.0225(105) | jump into the sea | 0.0194(40) |
| burn charcoal | 0.3406  (888) | don’t want | 0.0559(268) | private conversation | 0.0292(26) | hope | 0.0217(103) | private chat | 0.0191(37) |
| hang | 0.1546  (385） | have or not | 0.0559(224) | depression | 0.0266(89) | choose | 0.0208(113) | self-harm | 0.0191(49) |
| really | 0.1218(592) | alive | 0.0463(171) | afraid | 0.0262(107) | ugly | 0.0204(57) | get on the way | 0.0190(67) |
| jump into a river | 0.1178(253) | feel | 0.0427(200) | thanks | 0.0251(35) | death | 0.02039(86) | method | 0.0185(106) |
| jump down | 0.1005(286) | uncomfortable | 0.0378(131) | relieve | 0.0248(73) | mama | 0.0202(83) | family | 0.0180(68) |
| suicide | 0.0920(366) | taking pills | 0.0345(109) | happy | 0.0246(86) | like | 0.0201(94) | think | 0.0167(66) |
| pain | 0.0840(328) | afraid of pain | 0.0321(69) | be like | 0.0239(119) | pass away | 0.0200(72) | discover | 0.0164(95) |
| sleeping pills | 0.0729(189) | sad | 0.0316(121) | fail to die | 0.0236(29) | I will | 0.0198(74) | very painful | 0.0163 (30) |
